# Case Report: Lifesaving Hemostasis With Resuscitative Endovascular Balloon Occlusion of the Aorta in a Patient With Cardiac Arrest Caused by Upper Gastrointestinal Hemorrhage

**DOI:** 10.3389/fmed.2021.777421

**Published:** 2021-11-02

**Authors:** Tomoaki Hashida, Nanami Hata, Akiko Higashi, Yoshito Oka, Shunsuke Otani, Eizo Watanabe

**Affiliations:** ^1^Department of Emergency and Critical Care Medicine, Eastern Chiba Medical Center, Togane, Japan; ^2^Department of General Medical Science, Graduate School of Medicine, Chiba University, Chiba, Japan; ^3^Department of Emergency and Critical Care Medicine, Graduate School of Medicine, Chiba University, Chiba, Japan; ^4^Department of Surgery, Eastern Chiba Medical Center, Togane, Japan

**Keywords:** cardiopulmonary arrest, cardiopulmonary resuscitation, radiology, interventional, shock

## Abstract

Resuscitative endovascular balloon occlusion of the aorta (REBOA) is performed to treat hemorrhagic shock, whose cause is located below the diaphragm. However, its use in patients with gastrointestinal hemorrhage is relatively rare. The 45-year-old man with a history of dilated cardiomyopathy had experienced epigastric discomfort and had an episode of presyncope. On his presentation, the patient's blood pressure was 82/64 mmHg, heart rate 140/min, and consciousness level GCS E4V5M6. Hemodynamics stabilized rapidly with a transfusion that was administered on an emergency basis, and a blood sample only showed mild anemia (Hb, 11.5 g/dL). The patient was admitted to investigating the presyncope episode, and the planned endoscopy was scheduled the following day. The patient had an episode of presyncope soon and was found in hemorrhagic shock resulting from a duodenal ulcer rapidly deteriorated to cardiac arrest. Although a spontaneous heartbeat was restored with cardiopulmonary resuscitation, the patient's hemodynamics were unstable despite the emergency blood transfusion administered by pumping. Consequently, a REBOA device was placed, resuscitation was continued, and hemostasis was achieved by vascular embolization for the gastroduodenal artery. The patient was subsequently discharged without complications. However, there is no established evidence regarding the REBOA use in upper gastrointestinal hemorrhage, and the investigations that have been reported have been limited. Further, one recent research suggests that appropriate patient selection and early use may improve survival in these life-threatening cases. As was seen in the present case, REBOA can effectively treat upper gastrointestinal hemorrhage by temporarily stabilizing hemodynamics and enabling a hemostatic procedure to be quickly performed during that time. This report also demonstrated the hemodynamics during the combination of intermittent and partial REBOA to avoid the complications of ischemic or reperfusion injury of the intestines or lower extremities.

## Introduction

REBOA is performed to treat hemorrhagic shock whose cause is located below the diaphragm, and its use in trauma and obstetrical hemorrhage has been frequently reported (1–3). However, its use in patients with gastrointestinal hemorrhage is relatively rare (4, 5). Although endoscopic hemostasis is the first-line option for upper gastrointestinal hemorrhage (6), vascular embolization or laparotomy is necessary if the patient's hemodynamics are unstable or if bleeding cannot be controlled, making visualization difficult. This report describes a case in which a patient who went into cardiac arrest due to hemorrhagic shock resulting from a duodenal ulcer was saved by hemostasis achieved by vascular embolization performed in combination with REBOA.

## Case Description

Patient: The patient was a 45-year-old man with a body mass index of 19.8 kg/m^2^.

Past history: Dilated cardiomyopathy and placement of a cardiac resynchronization therapy-defibrillator (CRTD).

Oral medication history: Antihypertensive drug, diuretic.

During the previous several months, the patient had experienced epigastric discomfort. He had an episode of presyncope after a meal and was transported to our hospital by ambulance. On presentation, the patient's blood pressure was 82/64 mmHg, heart rate 140/min, SpO_2_ 98% (room air), and consciousness level GCS E4V5M6. Ejection fraction (EF) was determined on an echocardiogram and found to be 20%. Although tachycardia and decreased blood pressure were seen, hemodynamics stabilized rapidly with a transfusion of <1,000 mL fluid that was administered on an emergency basis. A blood sample showed mild anemia (Hb, 11.5 g/dL) but no other significant findings. Although contrast computed tomography (CT) showed an irregularity in part of the wall of the descending portion of the duodenum, no perforation or extravasation was seen. There were no other significant findings in the head or chest. The patient was admitted to thoroughly investigate the presyncope episode, and the planned endoscopy was scheduled the following day.

Although the patient's vital signs were stable after admission, blood collected on the day after admission showed worsening anemia (Hb, 7.6 g/dL). Duodenal hemorrhage was suspected based on the patient's clinical course, and upper gastrointestinal endoscopy was scheduled the following day due to gastric fullness (Supplementary Figure 1). However, the patient had an episode of presyncope before the endoscopic examination was performed, and an emergency call was placed in the general ward. When examined by the physician, the patient was found to be in shock, and emergency transfusion was started immediately. However, the patient went into cardiac arrest. Although a spontaneous heartbeat was restored with cardiopulmonary resuscitation, the patient's hemodynamics were unstable. Because a large volume of bloody drainage was seen from the nasogastric tube that had been inserted, the cause of the cardiac arrest was concluded to be upper gastrointestinal hemorrhage, and a decision was made to perform emergency hemostasis. However, the emergency blood transfusion administered by pumping barely produced pulse pressure. Consequently, there was not sufficient time to proceed to a CT examination. Placement of a REBOA device was therefore planned to temporarily stabilize the patient's hemodynamics.

**Figure 1:**
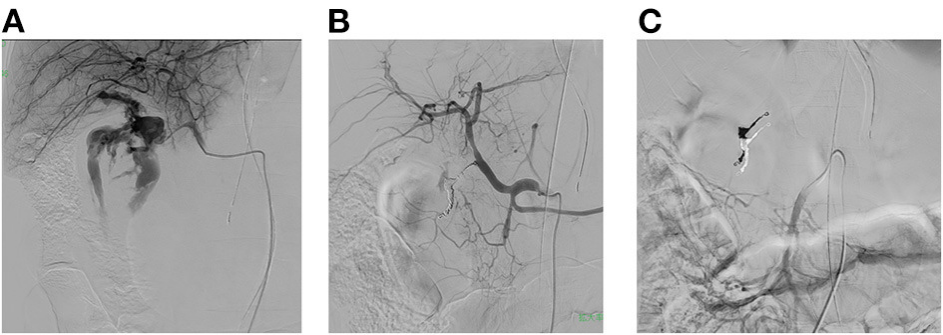
**(A)** Imaging is performed *via* the hepatic artery with the REBOA balloon inflated. A large volume of extravasation from the gastroduodenal artery is seen in the duodenum. **(B)** After completion of the embolization procedure, imaging is performed *via* the celiac artery with the REBOA balloon deflated. The gastroduodenal artery has been embolized with coils, and the extravasation has almost completely disappeared. **(C)** After completion of the embolization procedure, imaging is performed *via* the superior mesenteric artery with the REBOA balloon deflated. The ischemic area associated with embolization was small, and there were no significant complications.

The patient was moved to a fluoroscopy room as resuscitation was continued. A REBOA device (Rescue Balloon ER^®^, Tokai Medical Products, Kasugai, Japan) was then inserted *via* the left femoral artery and placed in zone I (region between the left subclavian artery and the celiac artery). Blood pressure increased transiently due to inflation of the REBOA balloon. Vascular embolization was planned as the hemostasis procedure, and it was determined that it would be performed in the fluoroscopy room. A 4-Fr sheath was placed contralaterally *via* the right femoral artery. Imaging was performed *via* the celiac and superior mesenteric arteries, and extravasation was seen from the gastroduodenal artery to within the duodenum (Figure 1A). Isolation of the hemorrhage site by coil embolization was then planned. The gastroduodenal artery was embolized using 11 ionization coils (Target Detachable Coil^®^, Stryker, Tokyo, Japan). During the embolization procedure, the transfusion rate was adjusted to manage hemodynamics as appropriate, while ischemia distal to the REBOA device was prevented by deflating the REBOA balloon. Angiography performed after embolization showed that the extravasation had nearly disappeared (Figure 1B). Moreover, no marked decrease in blood pressure was seen when the REBOA balloon was completely deflated. After completion of the embolization procedure, imaging is performed *via* the superior mesenteric artery with the REBOA balloon deflated. The ischemic area associated with embolization was confirmed to be small (Figure 1C), and there were no significant complications. The procedure was therefore completed, and the patient was returned to the intensive care unit (ICU). The transfusion required to maintain circulation from resuscitation to the end of the embolization procedure used 26 units of RBCs and 16 units of fresh frozen plasma (FFP).

**Figure 2:**
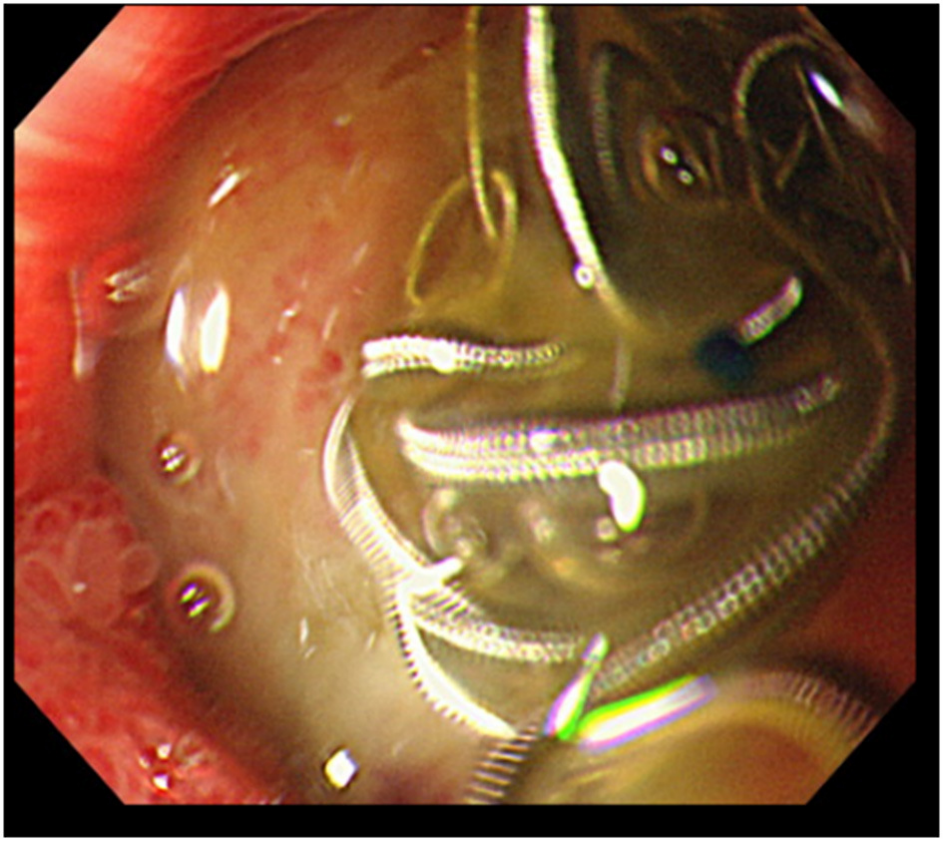
Upper gastrointestinal endoscopy imaging findings. Visible are the ulcer and leakage of the coils used to perform embolization in the anterior wall of the duodenal bulb.

After the patient returned to the ICU, there were no findings suggestive of recurrent bleeding, and hemodynamics were stable. Although the patient had a past history of dilated cardiomyopathy and was therefore weaned from mechanical ventilation carefully, mechanical ventilation was withdrawn without incident on day 8 of his illness. Upper gastrointestinal endoscopy performed on day 11 of hospitalization showed an ulcer in the anterior wall of the duodenal bulb and coil extrusion but no bleeding (Figure 2). Oral food intake was subsequently started, and the patient was able to walk without assistance at discharge and had no neurological sequelae.

## Discussion

By performing vascular embolization in combination with REBOA, it was possible to resuscitate and save the life of a patient who went into cardiac arrest as a result of upper gastrointestinal hemorrhage. REBOA has been used mainly for resuscitation in hemorrhagic shock that results from trauma and whose cause is located below the diaphragm. The benefits of REBOA are that it can enable a bridge to a hemostatic procedure while providing temporary hemostasis and control of blood loss volume and maintaining blood flow to the brain and heart (1, 2). In addition, there have been numerous reports of its effectiveness in cases that do not involve trauma, such as critical obstetrical hemorrhage and aortic aneurysm rupture (3, 7). However, there is no established evidence regarding its use in upper gastrointestinal hemorrhage, and the investigations that have been reported have been limited to individual cases and facilities (4, 5). Further, one recent research suggests that appropriate patient selection and early use may improve survival in these life-threatening cases (8). As was seen in the present case, REBOA can effectively treat upper gastrointestinal hemorrhage by temporarily stabilizing hemodynamics and enabling a hemostatic procedure to be quickly performed during that time.

**Figure 3:**
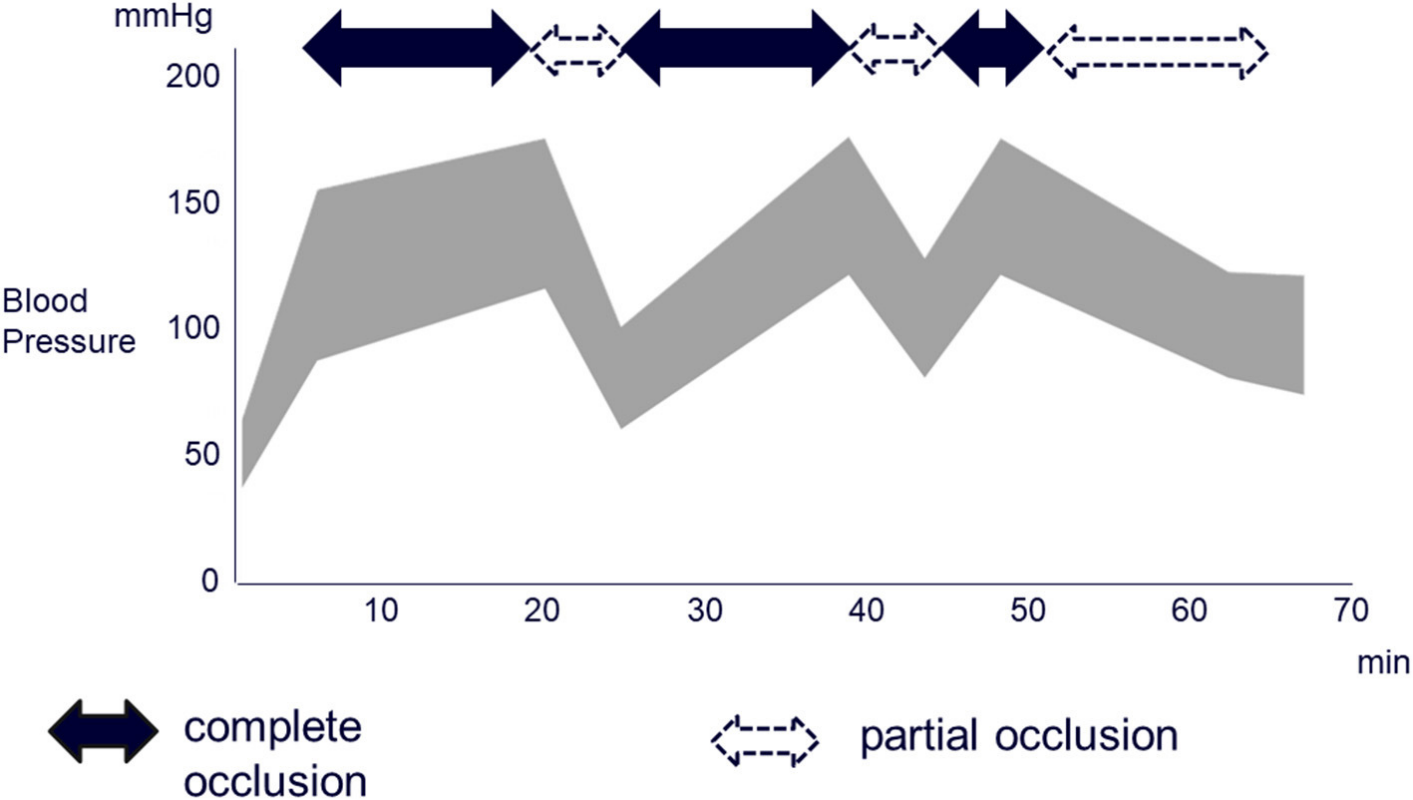
Changes in blood pressure over time after the patient were moved to the fluoroscopy room and the REBOA device was placed. In the complete and incomplete occlusions, balloon inflation volumes of REBOA were 25 and 15 mL, respectively.

Because the aorta is occluded for an extended period with REBOA, intestinal and lower extremity ischemia may occur (9). Another potential problem is that reperfusion injury may occur when blood flow is restored (9). Consequently, significantly reducing the volume of blood loss and minimizing ischemia-reperfusion injury by alternately inflating and deflating the balloon (intermittent REBOA) have been reported to improve survival (10). Moreover, partial occlusion of the aorta (partial REBOA) has been reported to be effective because it reduces ischemia distal to the occluded area moderately and can also reduce cardiac afterload proximal to the occlusion (11). In the present case as well, the REBOA device was placed, and the aorta was totally occluded initially to temporarily stabilize the circulation, and vascular embolization was then started. In addition, a transfusion was administered in a manner that ensured a sufficient supply, and total and partial occlusion were repeated in alternation for 15 and 5 min, respectively. Once the embolization proceeded to a certain point, the REBOA balloon was gradually deflated. The balloon was completely deflated after the embolization procedure was completed. The total duration of complete occlusion was 35 min, and the total duration of partial occlusion was 37 min (Figure 3). Because the event was managed by combining intermittent and partial REBOA to avoid complications, there was no ischemic or reperfusion injury of the intestines or lower extremities despite the relatively long period of REBOA use. The former study using a porcine hemorrhagic shock model reported that the complete aorta occlusion for more than 40 min caused severe complications (12). Therefore, the combination of intermittent and partial REBOA is a promising strategy for the severest hemorrhagic shock as the present case. To the best of our knowledge, this case report first demonstrated the hemodynamics during the combination of intermittent and partial REBOA to avoid the complications of ischemic or reperfusion injury of the intestines or lower extremities.

When upper gastrointestinal hemorrhage is suspected, and hemodynamics are relatively stable, the first-line option is to perform upper gastrointestinal endoscopy to identify and treat the source of the hemorrhage (6). In the present case, however, a large volume of bloody drainage was seen from the nasogastric tube, and visualization may therefore have been difficult even if endoscopy had been performed. In addition, contrast CT performed the previous day showed a mural irregularity in the descending portion of the duodenum, and pancreaticoduodenectomy, therefore, had to be considered as a laparotomy procedure. However, the patient's past history of dilated cardiomyopathy raised the possibility that surgery would not be well-tolerated. Moreover, hemodynamics were highly unstable, making it difficult to repeatedly move the patient. Because the patient was in the fluoroscopy room for REBOA device placement, the hemorrhage site could be identified by angiography without the risk of moving the patient, and vascular embolization was selected as the hemostasis procedure and completed successfully. Although the lesion turned out to be located in the duodenal bulb, hemostasis was performed by vascular embolization in combination with REBOA, and the patient's life was saved without complications.

## Conclusion

The patient went into cardiac arrest as a result of upper gastrointestinal hemorrhage, and resuscitation was performed in combination with REBOA. Vascular embolization was performed while repeatedly alternating between complete and partial occlusion, and the patient's life was saved.

This case suggests that REBOA may be an effective treatment strategy not only for hemorrhagic shock resulting from trauma but also for life-threatening upper gastrointestinal hemorrhage.

## Data Availability Statement

The original contributions presented in the study are included in the article/Supplementary Material, further inquiries can be directed to the corresponding author/s.

## Ethics Statement

Written informed consent was obtained from the individual(s), and minor(s)' legal guardian/next of kin, for the publication of any potentially identifiable images or data included in this article.

## Author Contributions

TH, NH, AH, YO, SO, and EW contributed to the clinical care and discussion. TH and EW wrote the paper. All authors contributed to the article and approved the submitted version.

## Conflict of Interest

The authors declare that the research was conducted in the absence of any commercial or financial relationships that could be construed as a potential conflict of interest.

## Publisher's Note

All claims expressed in this article are solely those of the authors and do not necessarily represent those of their affiliated organizations, or those of the publisher, the editors and the reviewers. Any product that may be evaluated in this article, or claim that may be made by its manufacturer, is not guaranteed or endorsed by the publisher.

## Supplementary Material

Supplementary Figure 1 The abdominal CT finding of gastric fullness on admission.
