# Supplementary figures and images for: Case Report: Lifesaving Hemostasis With Resuscitative Endovascular Balloon Occlusion of the Aorta in a Patient With Cardiac Arrest Caused by Upper Gastrointestinal Hemorrhage
Source: Front Med (Lausanne). 2021 Nov 2;8:777421. doi: 10.3389/fmed.2021.777421 (PMC8592922; doi:10.3389/fmed.2021.777421)

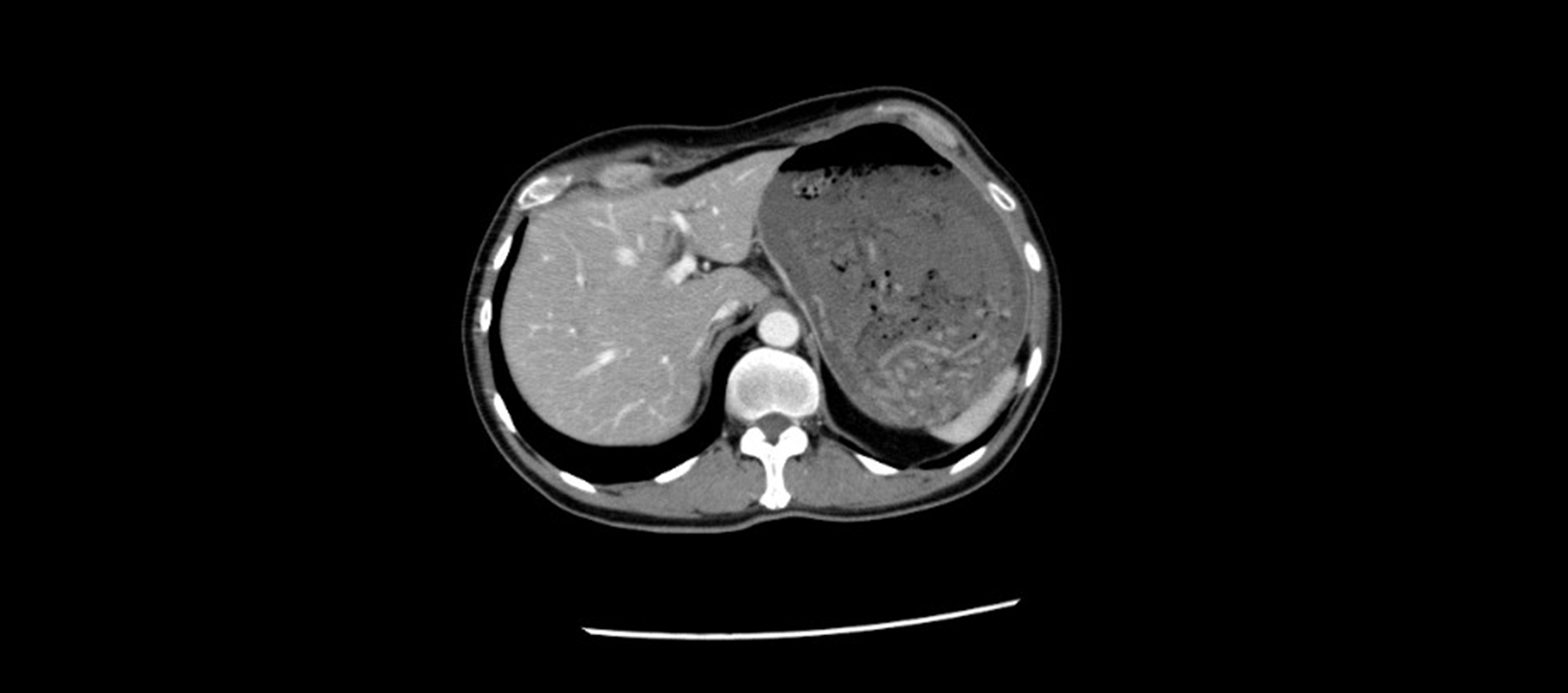

Supplement: Supplementary Figure 1 — The abdominal CT finding of gastric fullness on admission. [file Image_1.JPEG]
